# Dermoscopy of nailfold capillaries and Gottron's papules in a 15-year-old patient

**DOI:** 10.1016/j.jdcr.2024.12.038

**Published:** 2025-02-27

**Authors:** Ludwin Castro, Manuel Valdebran, Katherine G. Meléndez, Johanna Cristales, Luz Ely Cerritos

**Affiliations:** a Department of Dermatology, Zacamil National Hospital, San Salvador, El Salvador; b Department of Dermatology, Medical University of South Carolina, Charleston, South Carolina

**Keywords:** dermatomyositis, dermoscopy, Gottron´s papules, nailfold capillaries, pediatrics

## Clinical presentation

A 15-year-old female patient with an 8-month history of juvenile dermatomyositis was referred from the rheumatology department to dermatology. Over the past month, she developed progressively erythematous papules on the dorsum of the hands, with mild pruritus. Examination revealed the presence of Gottron's papules.

## Dermatoscopic appearance

**Fig 1:**
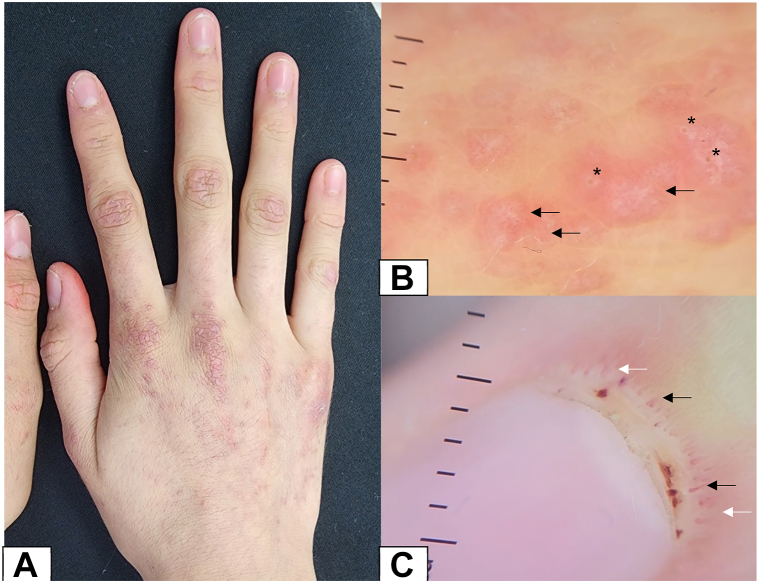
**A,** Clinical presentation of Gottron's papules on the dorsum of the hand over the joints. **B,***White* structureless areas on *pink* structureless areas and keratotic plugs on the dorsum of the hands. **C,** Enlarged capillaries, capillary loss, and cuticle hemorrhages in the proximal nailfold.

Using pocket DL200 Hybrid (DermLite) (magnification: ×20), white structureless areas on pink structureless areas (Black arrows) and keratotic plugs (Asterisk) were observed on the dorsum of the hands (Fig 1, *B*). In the proximal nailfolds, enlarged capillaries (Black arrows), capillary loss (White arrows) and cuticle hemorrhages (Asterisk) were noted (Fig 1, *C*).

**Key message**

As pathognomonic skin features, Gottron's papules and Gottron's sign, are described in dermatomyositis, Gottron's papules are erythematous skin lesions that are slightly infiltrated and purple, primarily on the metacarpophalangeal, interphalangeal, and distal interphalangeal joints.^1^ In most of the cases, Gottron's papules in dermatomyositis displayed pleomorphic vasculature (dotted and linear with branching or linear curved), white scales mostly dispersed unevenly, follicular plugs visible, white or pink structureless regions, red dots/globules corresponding to punctuate hemorrhages.^2^ Bushy capillaries are the most frequently observed dermoscopic feature of dermatomyositis, followed by avascular areas.^3^ Dermoscopic findings contribute to the diagnosis and monitoring of patients with dermatomyositis, primarily through its pathognomonic signs. This case report presents the dermoscopy of the nailfold and Gottron's papules in a patient with juvenile dermatomyositis.

## Conflicts of interest

None disclosed.
